# Incomplete Charge Collection at Inter-Pixel Gap in Low- and High-Flux Cadmium Zinc Telluride Pixel Detectors

**DOI:** 10.3390/s22041441

**Published:** 2022-02-13

**Authors:** Antonino Buttacavoli, Fabio Principato, Gaetano Gerardi, Donato Cascio, Giuseppe Raso, Manuele Bettelli, Andrea Zappettini, Paul Seller, Matthew C. Veale, Leonardo Abbene

**Affiliations:** 1 Department of Physics and Chemistry (DiFC)—Emilio Segrè, University of Palermo, Viale Delle Scienze, Edificio 18, 90128 Palermo, Italy; antonino.buttacavoli@unipa.it (A.B.); fabio.principato@unipa.it (F.P.); gaetano.gerardi@unipa.it (G.G.); donato.cascio@unipa.it (D.C.); giuseppe.raso@unipa.it (G.R.); 2 IMEM/CNR, Parco Area delle Scienze 37/A, 43100 Parma, Italy; manuele.bettelli@imem.cnr.it (M.B.); andrea.zappettini@imem.cnr.it (A.Z.); 3 Rutherford Appleton Laboratory, UKRI Science & Technology Facilities Council, Oxon OX11 0QX, UK; paul.seller@stfc.ac.uk (P.S.); matthew.veale@stfc.ac.uk (M.C.V.)

**Keywords:** CZT detectors, charge sharing, incomplete charge collection, charge-sharing correction, semiconductor pixel detectors

## Abstract

The success of cadmium zinc telluride (CZT) detectors in room-temperature spectroscopic X-ray imaging is now widely accepted. The most common CZT detectors are characterized by enhanced-charge transport properties of electrons, with mobility-lifetime products μeτe > 10^−2^ cm^2^/V and μhτh > 10^−5^ cm^2^/V. These materials, typically termed low-flux LF-CZT, are successfully used for thick electron-sensing detectors and in low-flux conditions. Recently, new CZT materials with hole mobility-lifetime product enhancements (μhτh > 10^−4^ cm^2^/V and μeτe > 10^−3^ cm^2^/V) have been fabricated for high-flux measurements (high-flux HF-CZT detectors). In this work, we will present the performance and charge-sharing properties of sub-millimeter CZT pixel detectors based on LF-CZT and HF-CZT crystals. Experimental results from the measurement of energy spectra after charge-sharing addition (CSA) and from 2D X-ray mapping highlight the better charge-collection properties of HF-CZT detectors near the inter-pixel gaps. The successful mitigation of the effects of incomplete charge collection after CSA was also performed through original charge-sharing correction techniques. These activities exist in the framework of international collaboration on the development of energy-resolved X-ray scanners for medical applications and non-destructive testing in the food industry.

## 1. Introduction

Nowadays, cadmium zinc telluride (CdZnTe or CZT) detectors have reached an excellent maturity level in room-temperature X-ray and gamma-ray detection, from photon energies of a few keV up to 1 MeV [1,2,3,4,5,6,7,8,9,10,11]. After the first CZT detector was presented by Butler in 1992 [12], intense research activities started with important progress made in both crystal growth and electrical contact technology. CZT detectors with pixel and strip electrodes were widely developed for X-ray and gamma-ray spectroscopic imaging, meeting the different requirements of many applications, including diagnostic-nuclear medicine [4,5,6,7], astrophysics [8,9,10,11], security [1], and non-destructive testing in the food industry [13,14]. Typically, the best-spectroscopic-grade CZT crystals are grown via Bridgman (B) [15,16,17,18] and traveling heater method (THM) growth [18,19,20,21,22] techniques. In particular, important progress in the charge carrier transport properties and uniformity in THM-CZT crystals has been made. The development of THM-CZT detectors with high electron transport properties, i.e., characterized by mobility-lifetime products of electrons *μ_e_τ_e_* greater than 10^−2^ cm^2^/V, was pioneered by Chen in 2007 [21]. Since then, several suppliers (Redlen Technologies, Canada; Kromek, UK) were able to fabricate high-*μ_e_τ_e_* CZT crystals with thicknesses greater than 10 mm. Excellent performances were obtained in thick CZT detectors (>5 mm), where the particular electron-sensing design (a drift strip and coplanar grids) is often optimized to work in low-electricity fields (<1000 V/cm) [8,9]. Besides this, great efforts have also been made to enhance the mobility-lifetime products of the holes (*μ_h_τ_h_*), especially for high-flux measurements. Enhancements in hole charge transport properties are necessary to minimize the effects of radiation-induced polarization phenomena at high fluxes [23,24,25,26]. Recently, high-*μ_h_τ_h_* THM-CZT crystals (*μ_h_τ_h_ >* 10^−4^ cm^2^/V) are provided by Redlen for high-flux applications [27,28,29]. Therefore, high-μ_h_*τ_h_* CZT detectors (high-flux HF-CZT detectors) are typically used for high-flux measurements, while high-*μ_e_τ_e_* CZT materials (low flux LF-CZT) for thick electron-sensing detectors generally work at low flux conditions. 

As is well known, the most important critical issues in sub-millimeter CZT pixel detectors are represented by charge-sharing and crosstalk effects, with the presence of severe degradations in both the spectral and spatial performance [29,30,31,32,33,34,35,36,37,38,39,40]. Despite the potential of charge-sharing addition (CSA) techniques in reducing the charge-sharing effects, the presence of incomplete charge collection in the summed energy after CSA often prevents full energy recovery in the energy spectra [33,34,35,36,37,38,39,40]. These effects, due to the presence of charge losses at the inter-pixel gaps, are strictly related to the physical properties of the detectors (crystals and electrical contacts) near the gaps between the pixels. 

In this work, we investigated the effects of charge losses near the inter-pixel gap in LF/HF-THM CZT pixel detectors. The detectors are fabricated on THM-CZT crystals recently produced by Redlen Technologies company (Canada), for both low- (LF-CZT) [19,20,21,22] and high- (HF-CZT) flux applications [27,28,29]. Measurements with uncollimated radiation sources and collimated synchrotron X-ray beams were performed on LF/HF-CZT pixel detectors characterized by the same crystal dimensions, electrical layout, bias voltage operation, and readout electronics.

## 2. The CZT Pixel Detectors 

**Figure 1:**
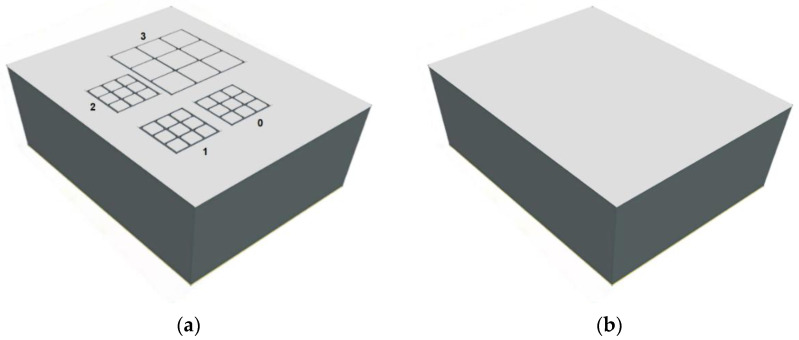
(**a**) The anode layout of the CZT pixel detectors. Array 3 is characterized by a pixel pitch of 500 µm, while the other three arrays by a pixel pitch of 250 µm. The width of the inter-pixel gaps is equal to 50 µm for all arrays. All arrays are surrounded by a guard-ring electrode up to the edge of the crystal. (**b**) The cathode electrode covers the surface of the CZT crystals. The figures are not drawn to scale.

Two-millimeter-thick CZT pixel detectors characterized by the same electrode layout and dimensions (4.25 × 3.25 × 2 mm^3^) were investigated. Regarding the electrode layout (Figure 1), all anodes consist of four arrays of 3 × 3 pixels: a large array with a pixel pitch of 500 µm and three small arrays with pixel pitches of 250 µm. 

The cathode is a simple planar electrode covering the surface of the detectors. All arrays are designed with the same inter-pixel gap of 50 µm. The detectors are based on low-flux LF-THM CZT and high-flux HF-THM CZT crystals, developed by Redlen Technologies (Canada).

### 2.1. Low Flux LF-CZT Detectors

The LF-CZT pixel detectors were fabricated at IMEM-CNR of Parma (Parma, Italy) in collaboration with the due2lab company (Reggio Emilia, Italy). The detectors are based on THM-CZT crystals provided by Redlen and characterized by gold electro-less contacts. Recently, very-low-noise gold contacts were realized on CZT detectors by our group [19,35,36,37,38,41], ensuring low leakage currents at room temperature (4.7 nA cm^−2^ at 1000 V cm^−1^) and good room-temperature operation even at high bias voltages (> 5000 V cm^−1^). The LF-CZT detectors are characterized by *μ_e_τ_e_* ranging from 1 to 3 × 10^−2^ cm^2^/V and *μ_h_τ_h_* from 2 to 3 × 10^−5^ cm^2^/V [27]. 

### 2.2. Hih Flux HF-CZT Detectors

The HF-CZT pixel detectors were fabricated by Redlen Technologies with platinum electrical contacts. The charge transport properties are represented by *μ_e_τ_e_* ranging from 2 to 3·10^−3^ cm^2^/V and *μ_h_τ_h_* from 1 to 2·10^−4^ cm^2^/V [27].

### 2.3. Readout Electronics

The processing of the detector signals was performed using the same readout electronics for all detectors. Regarding the front-end electronics, the detectors were flip-chip bonded to charge-sensitive preamplifiers (CSPs) based on a low-noise application-specific integrated circuit (PIXIE ASIC) developed at RAL (Didcot, UK) [42]. The PIXIE ASIC, characterized by an equivalent noise charge (ENC) less than 80 electrons, provides nine output channels at the same time for each 3 × 3 pixel array. The output waveforms from the PIXIE ASIC are digitized and processed online by 16-channel digital electronics, developed at DiFC of the University of Palermo (Italy) [35,36,43,44]. The digital electronic is based on commercial digitizers (DT5724, 16-bit, 100 MS/s, CAEN SpA, Italy), where an original firmware was uploaded [43,44]. 

### 2.4. Spectroscopic Performance 

**Figure 2:**
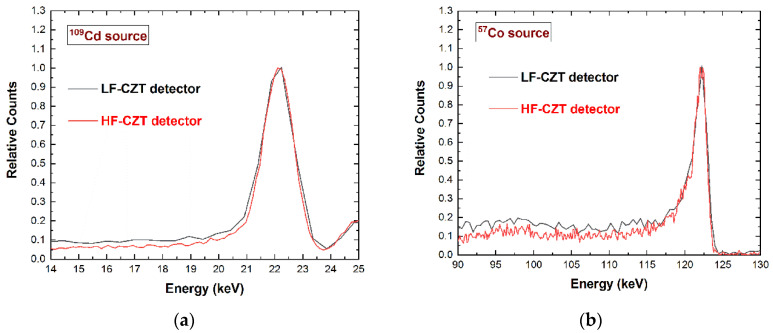
Measured energy spectra of a pixel of the 500 μm array for the LF-CZT (black line) and HF-CZT (red line) detectors. (**a**) The energy resolution of the main peaks (22.1 keV) of ^109^Cd energy spectra is approximately 6% (1.3 keV) FWHM for both detectors. (**b**) The 122 keV peaks of the ^57^Co spectra are characterized by an energy resolution of 1.7% (2 keV). To compare the photopeak shapes, the energy spectra are normalized to the photopeak centroid counts.

The spectroscopic response of the detectors, when illuminated with uncollimated radiation sources (^109^Cd, ^241^Am, ^57^Co), was measured. The detectors, biased at 1000 V (5000 V/cm), are characterized by a similar room-temperature performance (T = 20 °C), as shown in Figure 2. This bias voltage value represents, for both detectors, the optimum setting for the best energy resolution. The energy resolution (FWHM) of both detectors is from less than 2 keV up to 122 keV. The similar performance of the detectors, despite the different charge transport properties of the CZT crystals, is due to several factors, e.g., the high-bias voltage operation, the small thickness, and the electron-sensing properties of the pixel detectors. 

## 3. Incomplete Charge Collection in Charge-Sharing Events

Charge-sharing measurements were performed through time coincidence analysis, with a particular focus on the coincidence events of the central pixel of each array with the neighboring pixels. The coincidence events were detected within a coincidence time window (CTW) of 200 ns, ensuring the full detection of all coincidence events. The number of coincidence events is the same for both detectors, confirming its dependence on the detector layout geometry. The percentage of the coincidence events is very high, and this number increases for high values of the ratio between the inter-pixel gap area and the pixel area and for energies greater than the K-shell absorption energy of the CZT material (26.7 keV, 9.7 keV, and 31.8 keV for Cd, Zn, and Te, respectively).

**Figure 3:**
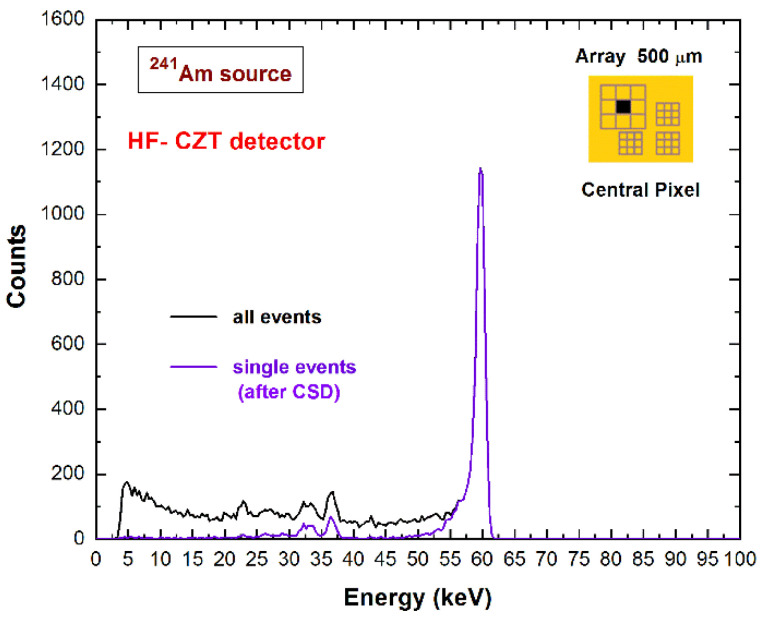
Comparison between the raw ^241^Am energy spectrum (black line) and the spectrum (violet line) after charge-sharing discrimination (CSD). The energy spectra are related to the central pixel of the 500 μm array of the HF-CZT detector. After CSD, 56% of all events are rejected.

**Figure 4:**
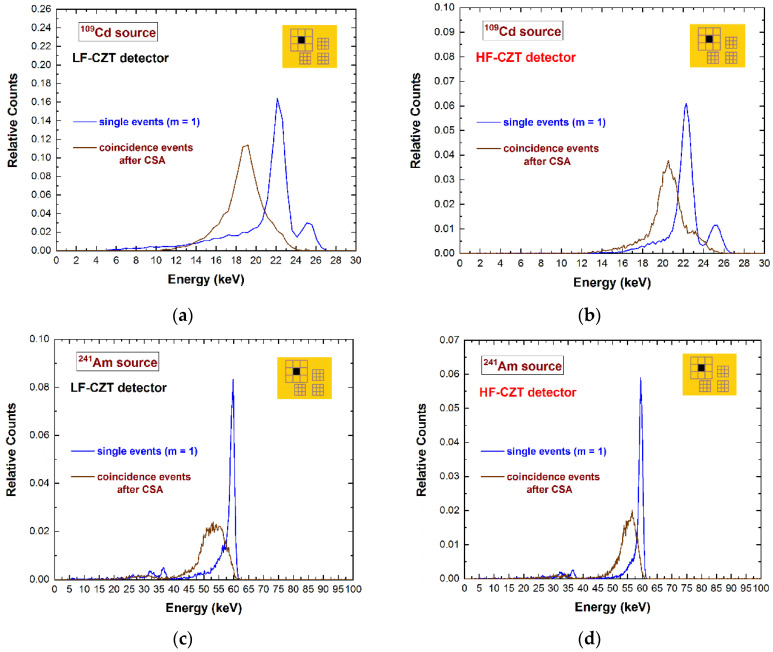
Measured ^109^Cd and ^241^Am energy spectra after the application of the charge-sharing addition (CSA) technique. The energy spectra of the single events (blue lines) and the spectra of the coincidence events with the eight adjacent pixels (multiplicity *m* = 2) after CSA (brown lines) for the (**a**), (**c**) LF-CZT and (**b**), (**d**) HF-CZT detectors. The energy spectra after CSA are characterized by energy deficits due to the presence of charge losses near the inter-pixel gaps, more severe for the LF-CZT detectors.

**Figure 5:**
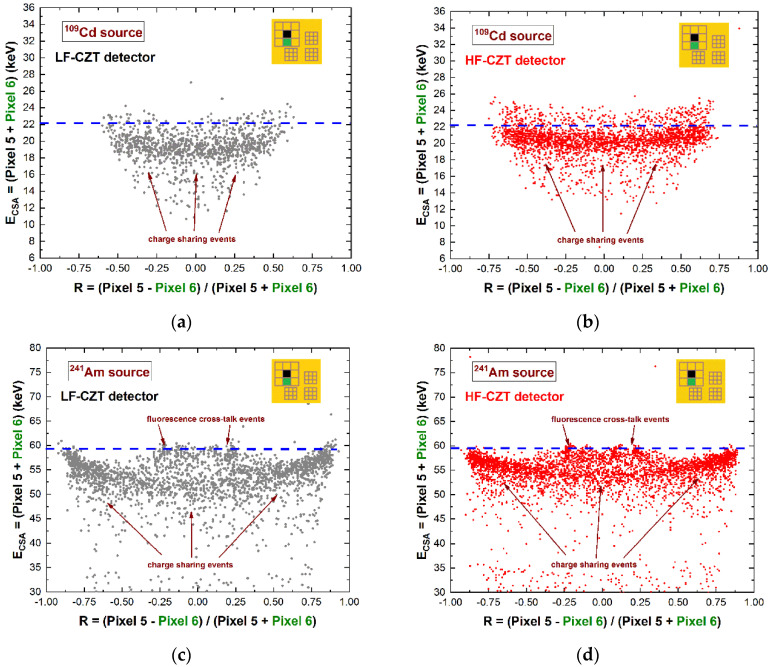
Two-dimensional (2D) scatter plot of the summed energy *E_CSA_* of the coincidence events (*m* = 2) between two adjacent pixels (central pixel no. 5 and pixel no. 6), after CSA, versus the charge-sharing ratio *R*. The plots highlight the dependence of the energy deficit on the position within the inter-pixel gap and the higher charge losses of (**a**), (**c**) the LF-CZT than (**b**), (**d**) the HF-CZT detector.

In this last case, the fluorescent X-rays create both an increase in the charge cloud dimensions and the generation of crosstalk phenomena between the pixels. Regarding the coincidence percentages, the results for the central pixel show values of 33–34% and 52–53% at 22.1 keV for the 500 µm and 250 µm arrays, respectively; while at energies greater than the K-shell absorption energy, we measured values of 56–58% and 79–81% at 59.5 keV for the 500 µm and 250 µm arrays, respectively. Figure 3 shows a comparison between the raw energy spectrum of the central pixel of the large 500 μm array (black line) and the spectrum with only single events (violet line), i.e., the events with multiplicity *m*= 1. The shape of the energy spectrum is strongly improved after the rejection of the coincidence events, i.e., after the application of the charge-sharing discrimination (CSD) technique. The coincidence events are mainly a result of charge-sharing and fluorescence crosstalk phenomena, and their effects are clearly visible in the raw spectrum: The fluorescent peaks at 23.2 and 27.5 keV, the escape peaks at 36.3 and 32 keV, the low-energy background, and tailing. As is well known, a typical recovery of the rejected events after CSD can be performed through the classical charge-sharing addition (CSA) technique. Figure 4 shows the results obtained after the application of CSA in LF/HF-CZT detectors, at energies below (^109^Cd source) and above (^241^Am source) the K-shell absorption energy of CZT. Both detectors are characterized by energy deficits in the spectra after CSA (brown lines), particularly of about 3.1 keV (6 keV) and 1.6 keV (3 keV) at 22.1 keV (59.5 keV) for the LF and HF CZT detectors, respectively. We also observed that this energy deficit is strongly related to the bias voltage, e.g., at 500 V, we measured a deficit of about 9 keV and 5 keV at 59.5 keV for the LF and HF detectors, respectively. Moreover, the distribution of these energy deficits within the inter-pixel gap between two pixels is also presented in Figure 5. The two-dimensional (2D) scatter plots show the summed energy *E_CSA_* of the coincidence events (*m = 2*) between two adjacent pixels (pixels no. 5 and no. 6), after CSA, versus the charge-sharing ratio *R*. The quantity *R*, calculated from the ratio between the energies of two adjacent pixels (Figure 5), gives indications about the position of the photon interaction between the two pixels.

**Figure 6:**
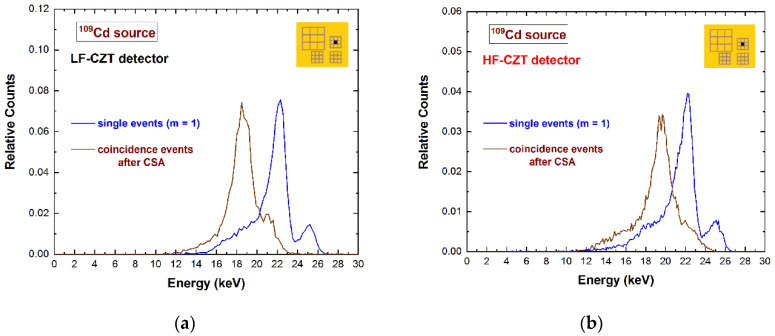
Measured ^109^Cd energy spectra with single events (blue line) and coincidence events (multiplicity *m* = 2) after CSA (brown line) for the (**a**) LF-CZT and (**b**) HF-CZT detectors. The energy deficits due to charge losses near the inter-pixel gaps are more severe for the LF-CZT detector.

The curvature shows the presence of energy deficits for both detectors and at all energies; they are more severe at *R* = 0, i.e., related to events interacting at the center of the inter-pixel gap. The summed energy *E_CSA_* of certain coincidence events at about *R* = 0.22 is fully recovered after CSA. These events represent the coupling of fluorescent X-rays at 23.2 keV and the escape events at 36.3 keV. As is clearly shown in Figure 4 and Figure 5, the energy deficit after CSA is less severe for the HF-CZT detectors. Recently, similar results were obtained by other researchers [29]; in particular, they measured less-incomplete charge collection after CSA in HF-CZT pixel detectors, in comparison with CdTe pixel detectors, attributing this to the better hole transport properties of the HF-CZT crystals than the CdTe ones. We believe that this difference is not dependent on the charge transport properties of the carriers but is strictly related to the characteristics of the electric field lines near the inter-pixel gaps. To confirm this conclusion, we also measured the energy deficits in charge-sharing events, where the charge clouds are mainly contributed to by the electrons; in this case, because of the better electron charge transport properties of the LF-CZT detectors than the HF-CZT ones, the charge losses in LF-CZT detectors should be less severe than the HF-CZT ones. In order to investigate shared events with high electron contributions in the charge cloud, we used low-energy X-rays (22.1 keV X-rays from the ^109^Cd source) interacting near the cathode side and measured the events from the pixels of the 250 μm arrays, characterized by high electron-sensing properties (in agreement with the small pixel effect [2]). The results are presented in Figure 6.

The energy deficits of the 22.1 keV photopeaks after CSA are approximately 3.5 keV and 2.5 keV for the LF-CZT and HF-CT detectors, respectively; the degradation continues to be more severe for the LF-CZT detector, confirming that the charge losses are not related to the transport properties of the charge carriers. This was also confirmed by simulation procedures. We performed a simulation of the two-dimensional (2D) scatter plots of the summed energy *E_CSA_* of the coincidence events (*m = 2*) between two adjacent pixels, for both detectors and arrays. The simulations were carried out by means of a first principle tool [45], composed of three main blocks: (i) The radiation–semiconductor interaction based on the Monte Carlo approach (Geant4), (ii) electric and weighting field calculation by the finite element method (FEM) with COMSOL Multiphysics, and (iii) the computation of the charge carrier transport and pulse formation in the MATLAB environment. In order to evaluate whether the charge losses in LF/HF CZT detectors are due to the transport properties of carriers, a spatially homogeneous distribution of charges was simulated inside the whole CZT volume. To focus the attention on the transport properties, any crystal defects (e.g., Te inclusions) or inter-pixel electric field distortions were implemented in simulations. The simulation was carried out using the physical quantities reported in Table 1.

**Figure 7:**
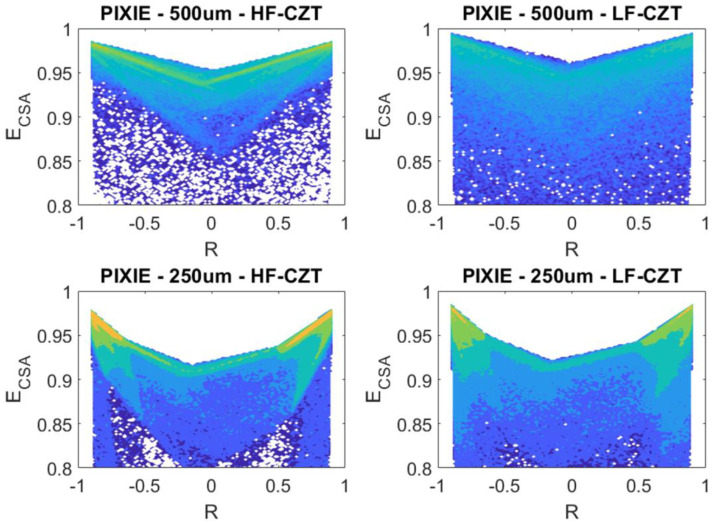
Simulated two-dimensional (2D) scatter plots of the summed energy E_CSA_ of the coincidence events (*m* = 2) between two adjacent pixels, after CSA, versus the charge-sharing ratio R obtained for 500 um and 250 um pitch pixel arrays and calculated for HF and LF−CZT.

We simulated the two-dimensional (2D) scatter plots of the summed energy E_CSA_ of the coincidence events (*m* = 2) between two adjacent pixels, after CSA, versus the charge-sharing ratio *R*, for both detectors and arrays (Figure 7). No remarkable differences are present in the comparison of the charge losses. This means that transport properties do not significantly influence the charge loss mechanism.

**Figure 8:**
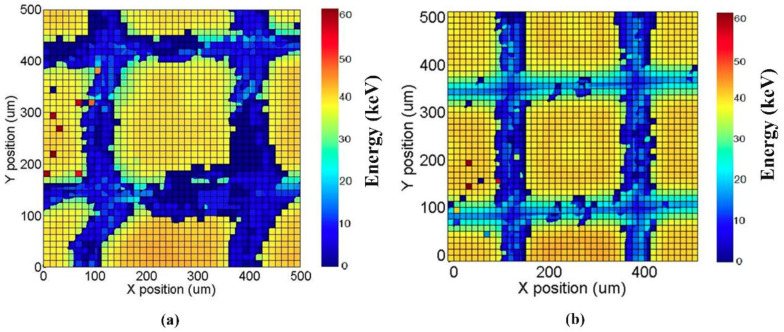
The results of a 2D synchrotron X-ray mapping of the small 250 μm array at 40 keV, for (**a**) the LF-CZT and (**b**) HF-CZT detectors. The changes in the photopeak energy are confined near the inter-pixel gaps because of charge-sharing effects, which are more severe for the LF-CZT detector.

These effects are due to the presence of distortions and non-uniformities in the electric field lines near the gaps between the pixels. This was also confirmed by the results obtained after a micro-scale 2-D X-ray mapping of the detectors. Collimated synchrotron X-ray beams were used at the B16 test beamline at the Diamond Light Source facility (Didcot, UK), in particular, 10 × 10 μm^2^ micro-beams with scan steps of 12.5 μm at 40 keV. The results of the mapping for the small 250 μm arrays are shown in Figure 8, where the 40 keV photopeak energy at different positions is presented. 

**Figure 9:**
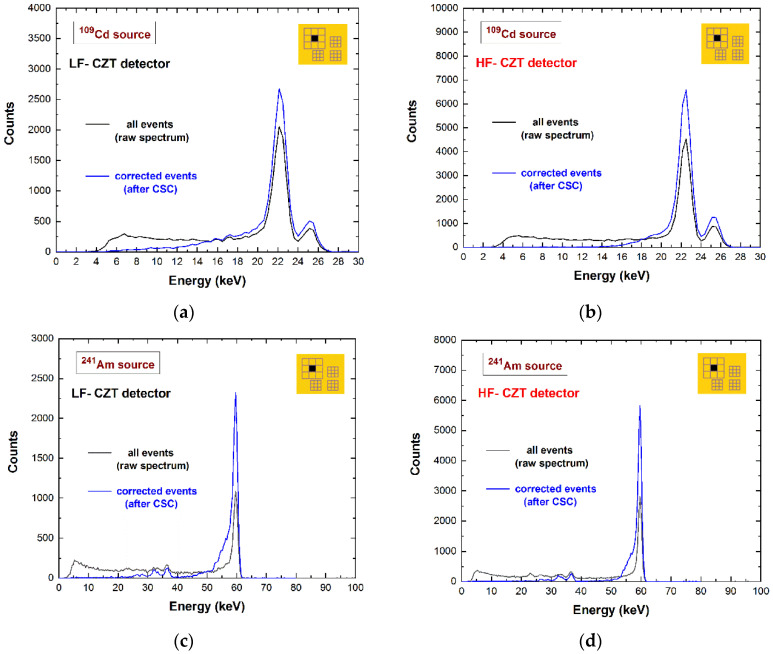
Spectroscopic results after the correction of the charge-sharing and fluorescence crosstalk events (after CSC). The raw energy spectra (black lines) and the corrected spectra (blue lines) for (**a**), (**c**) the LF-CZT and (**b**), (**d**) HF-CZT detectors are shown. The energy resolution of the HF-CZT detector was slightly improved after correction.

Both detectors, due to the charge sharing, show a reduction in the photopeak energy near the inter-pixel gaps; however, the LF-CZT detector is characterized by an extended charge-sharing region (blue region), due to the increased presence of distortions and non-uniformities in the electric field lines. The cause of these distortions is unclear but may be due to either the presence of crystalline defects (inclusions) or perhaps issues with the electrical contacts. Fortunately, the energy deficits of the shared events after CSA can be recovered through a custom correction technique, recently developed by our group [36,37]. This technique is based on the correction of (i) the shared events after CSA with *m* = 2, (ii) the events with *m* > 2, and (iii) the fluorescence crosstalk events. The results after charge-sharing correction (CSC) for both LF/HF-CZT detectors are presented in Figure 9.

As is clearly shown, the shared events are aptly detected and corrected in both detectors. However, while the raw and corrected spectra for the LF-CZT detector have the same energy resolution (1.8 keV FWHM at 59.5 keV), improvements in the energy resolution (1.4 keV versus 1.8 keV FWHM at 59.5 keV) characterize the corrected spectra for the HF-CZT detector. This result highlights the better collection properties of the HF-CZT detectors near the gaps between the pixels.

## 4. Conclusions

The spectroscopic performance and the charge-sharing properties of low-flux LF-THM CZT and high-flux HF-THM CZT pixel detectors are presented. The detectors, characterized by the same geometrical layout, allow high-bias voltage operation (5000 V/cm) and similar performances, with interesting room-temperature energy resolutions from < 2 keV FWHM up to 122 keV. Despite the same charge-sharing percentages between the pixels, different charge-collection properties in sharing events are observed, highlighted by the presence of different energy deficits in the spectra after CSA. The HF-CZT detectors show fewer energy deficits after CSA, due to their better charge-collection properties near the inter-pixel gap, as confirmed by the micro-scale X-ray mapping. These results can be justified by the presence of a better electric field line distribution near the inter-pixel gap in HF-CZT detectors. Finally, the result of our successful correction of these energy deficits in both detectors is presented. This shows dramatic improvements in both the energy recovery and the energy resolution for HF-CZT pixel detectors.

## Figures and Tables

**Table 1:** Simulation parameters.

High-Flux HF-CZT		Low-Flux LF- CZT	
$\mu_{e}\tau_{e}\;\left(cm^{2}/V\right)$	$2.5\times 10^{-3}$	$\mu_{e}\tau_{e}\;\left(cm^{2}/V\right)$	$2\times 10^{-2}$
$\mu_{h}\tau_{h}\;\left(cm^{2}/V\right)$	$1.5\times 10^{-4}$	$\mu_{h}\tau_{h}\;\left(cm^{2}/V\right)$	$2.5\times 10^{-5}$
Number of generated charges	$5\times 10^{6}$	Number of generated charges	$5\times 10^{6}$
Bias voltage (V)	1000	Bias voltage (V)	1000

## Data Availability

Not applicable.
